# Patients With Microscopic Colitis Have Altered Levels of Inhibitory and Stimulatory Biomarkers in Colon Biopsies and Sera Compared to Non-inflamed Controls

**DOI:** 10.3389/fmed.2021.727412

**Published:** 2021-10-15

**Authors:** Alexandra Lushnikova, Johan Bohr, Anna Wickbom, Andreas Münch, Klas Sjöberg, Olof Hultgren, Anders Wirén, Elisabeth Hultgren Hörnquist

**Affiliations:** ^1^School of Medical Sciences, Örebro University, Örebro, Sweden; ^2^Division of Gastroenterology, Department of Medicine, Örebro University Hospital, Faculty of Medicine and Health, Örebro University, Örebro, Sweden; ^3^Department of Gastroenterology and Hepatology in Linköping, and Department of Health, Medicine, and Caring Sciences, Linköping University, Linköping, Sweden; ^4^Department of Clinical Sciences, Lund University, Department of Gastroenterology, Skåne University Hospital, Malmö, Sweden; ^5^Department of Clinical Immunology and Transfusion Medicine, Faculty of Medicine and Health, Örebro University, Örebro, Sweden

**Keywords:** microscopic colitis, colorectal cancer, immune surveillance, immune checkpoints, ulcerative colitis, serum, colonic biopsies

## Abstract

**Introduction:** Microscopic colitis (MC) is an inflammatory bowel condition with two subtypes, lymphocytic colitis (LC) and collagenous colitis (CC). Unlike patients with ulcerative colitis (UC) and non-inflamed individuals, MC patients have reduced risk of developing colorectal cancer, possibly due to increased immune surveillance in MC patients.

**Aim:** To examine differences in levels of immunomodulatory molecules, including those involved in immune checkpoint mechanisms, in sera from patients with MC and in colonic biopsies from patients with MC and UC compared with controls.

**Methods:** Using Luminex, 23 analytes (4-1BB, 4-1BBL, APRIL, BAFF, BTLA, CD27, CD28, CD80, CTLA-4, E-cadherin, Galectin-3, GITR, HVEM, IDO, IL-2Rα, LAG-3, MICA, MICB, PD-1, PD-L1, PD-L2, sCD40L and TIM-3) were studied in serum from patients with active MC (*n* = 35) and controls (*n* = 23), and in colonic biopsies from patients with active LC (*n* = 9), active CC (*n* = 16) and MC in histological remission (LC *n* = 6, CC *n* = 6), active UC (*n* = 15) and UC in remission (*n* = 12) and controls (*n* = 58).

**Results:** In serum, IDO, PD-1, TIM-3, 4-1BB, CD27, and CD80 were decreased whereas 4-1BBL and IL-2Rα were increased in MC patients compared with controls. In contrast, in biopsies, levels of PD-L2 and 4-1BB were increased in MC and UC patients with active disease. Furthermore, in biopsies from CC and UC but not LC patients with active disease, CTLA-4, PD-1, APRIL, BAFF, and IL-2Rα were increased compared with controls. PD-L1 was increased in CC but not UC or LC patients. CD27 and TIM-3 were decreased in biopsies from MC patients in comparison to controls whereas levels of MICB were decreased in patients with active UC compared with controls.

**Conclusions:** Compared with non-inflamed controls, levels of soluble and membrane-bound immunomodulatory molecules were systemically and locally altered in MC and UC patients, with most analytes being decreased in serum but enhanced in colonic biopsies. These findings contribute to knowledge about checkpoint molecules and their role as biomarkers in MC and may also contribute to knowledge about possible mechanisms behind the seemingly protective effects of MC against colorectal cancer.

## Introduction

Microscopic colitis (MC) is an inflammatory bowel condition mostly affecting older women, consisting of two subtypes, lymphocytic colitis (LC) and collagenous colitis (CC). The incidence ranges from 7.2 to 20.7 per 100,000 person-years (1, 2). The etiology remains unknown, but proposed theories include a dysregulated immune response to unidentified luminal factors in genetically predisposed individuals (3, 4). Both subtypes share common symptoms, e.g., chronic watery, non-bloody diarrhea, abdominal pain, and weight loss. Upon endoscopy, no specific changes in the mucosa of MC patients are visible, therefore diagnosis is based on histological examination of colon biopsies. The diagnostic criteria for lymphocytic colitis include ≥20 intraepithelial lymphocytes (IELs) per 100 epithelial cells, whereas a subepithelial collagen layer ≥10 μm is required for diagnosis of collagenous colitis (3).

Patients with classical inflammatory bowel disease (IBD), i.e., Crohn's disease (CD) and ulcerative colitis (UC), have an increased risk of developing colorectal cancer (CRC) whereas MC patients instead display a decreased risk, even compared to the general population (5–8). The underlying mechanism remains unknown.

Immunomodulatory molecules, including the so-called immune checkpoints, regulate immune responses against possible threats, prevent inflammation from going into overdrive, and prevent autoimmunity. However, tumor cells can also take advantage of immune checkpoints and use them to downregulate T cell responses, leading to promotion of tumor survival (9). Immune surveillance describes the process by which malignant cells can be detected and eradicated and is just one component of the cancer immunoediting hypothesis that consists of three stages, namely elimination, equilibrium, and escape (10). A key feature of MC is an increased number of IELs, especially in LC, which may contribute to increased immune surveillance (11). This potentially increased immune surveillance could partly explain why MC patients are at lower risk of developing inflammation-associated CRC in comparison to UC patients and the general population (5–8).

Immune checkpoint inhibitors are utilized in the therapy of some cancers and help to reinvigorate intrinsic anti-tumor T cell responses (9). Immune-related adverse events, including colitis, are well-documented side effects of treatment with immune checkpoint inhibitors and some case reports have documented cases of MC following immune checkpoint inhibitor treatment. This suggests that immunomodulatory molecules play a key role in maintaining gut homeostasis (12–14).

Soluble forms of immunomodulatory molecules, produced through e.g., alternative mRNA splicing, have also been found to be important and investigation into whether serum levels can reflect disease progression and prognosis is ongoing (15, 16). Many immunomodulatory biomarkers, including immune checkpoints molecules, have been studied in classical IBD. For example, 4-1BB, a co-stimulatory receptor expressed on activated T cells, has been found to have higher expression on lamina propria T cells in CD patients compared to UC patients and controls (15, 17, 18). PD-1, expressed on activated T cells, exerts inhibitory effects through binding to its ligands PD-L1/PD-L2, and was found to be more highly expressed on lamina propria T cells of patients with IBD compared to controls (9, 19). Information about expression of molecules involved in immune checkpoints in patients with MC is, however, lacking.

As it remains unknown why MC patients display a decreased risk of developing CRC, studying immunomodulatory biomarkers including immune checkpoints can provide valuable insights into the underlying mechanisms at work. Our hypothesis is that patients with MC have increased immune surveillance, in contrast to patients with UC which seem to have an immune response that may lead to the development of inflammation-associated CRC. The aims of the present study were to study differences in levels of immunomodulatory biomarkers in sera from patients with microscopic colitis and in colonic biopsies from patients with microscopic colitis and ulcerative colitis in comparison to controls.

## Materials and Methods

Serum samples and biopsies were collected from two separate patient cohorts. Diagnosis of gastrointestinal disorders was made according to current diagnostic guidelines (3). MC disease activity (active/remission) was judged on clinical and histological evaluation. All MC patients in the present study had a confirmed histological diagnosis of MC. However, during our collection of the patient samples we found that some of these patients, despite having a previous histologically confirmed MC diagnosis and clinical symptoms, did not fulfill the histological criteria for MC at time of biopsy collection. These patients are therefore categorized as MC in histological remission and the results from these patients are presented as a separate group. The classification of UC patients into active disease or remission is based on combined evaluation of histological, endoscopic, and clinical factors.

### Patient Demographics

#### Serum Samples

**Figure 1:**
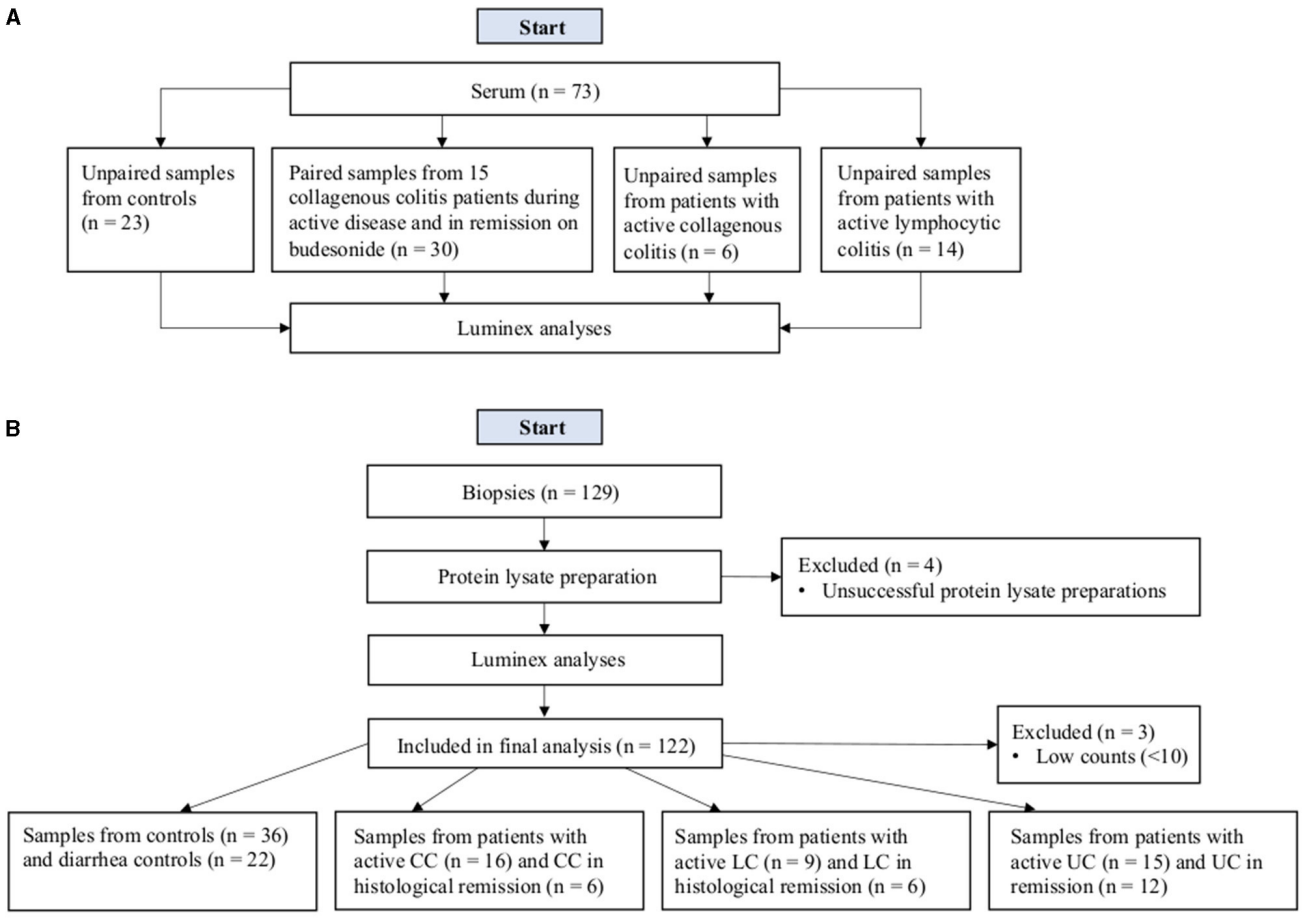
Flowchart of the study design for serum samples **(A)** and biopsies **(B)**.

In the serum group, 58 patients were included, of which 15 were CC patients where samples were obtained both during untreated, active disease and when in remission due to budesonide treatment, resulting in a total of 73 serum samples from patients with LC, CC, and controls (Figure 1A and Table 1). All controls had non-inflamed colons and the indications for medical investigation were diarrhea (*n* = 22) and fecal incontinence (*n* = 1). No patients with LC or CC received any immunomodulatory treatment at the time of serum sample collection apart from one patient with CC receiving betamethasone when necessary, for an indication unrelated to CC. All patients with CC in remission had been treated with budesonide for a duration of at least 6 weeks.

**Table 1:** Patient demographics for serum and colonic biopsy samples.

		**Sex:** ***n*** **(%)**			**Smoking**		
	**Diagnosis**	**Male**	**Female**	**Mean age (SD)**	**Yes *n* (%)**	**No** ***n*** **(%)**	**Unknown** ***n*** **(%)**
Serum	Control (*n =* 23)	5 (22)	18 (78)	58.3 (18.3)	4 (17)	17 (74)	2 (9)
	Active LC (*n =* 14)	1 (7)	13 (93)	59.1 (17.5)	2 (14)	11 (79)	1 (7)
	Active CC (*n =* 21)	4 (19)	17 (81)	58.8 (16.7)	7 (33)	13 (62)	1 (5)
	CC remission (*n =* 15)	4 (27)	11 (73)	55.7 (16.5)	5 (33)	10 (67)	0 (0)
Colonic biopsies	Control (*n =* 36)	20 (56)	16 (44)	60.0 (17.6)	8 (22)	18 (50)	10 (28)
	Diarrhea control (*n =* 22)	6 (27)	16 (73)	47.2 (17.9)	7 (32)	9 (41)	6 (27)
	Active LC (*n =* 9)	0 (0)	9 (100)	66.6 (8.2)	2 (22)	5 (56)	2 (22)
	LC-HR (*n =* 6)	0 (0)	6 (100)	56.7 (26.5)	2 (33)	4 (67)	0 (0)
	Active CC (*n =* 16)	2 (12.5)	14 (87.5)	56.2 (11.5)	4 (25.0)	10 (62.5)	2 (12.5)
	CC-HR (*n =* 6)	0 (0)	6 (100)	60.2 (9.0)	3 (50)	3 (50)	0 (0)
	Active UC (*n =* 15)	11 (73)	4 (27)	52.1 (15.5)	0 (0)	14 (93)	1 (7)
	UC remission (*n =* 12)	7 (58)	5 (42)	61.4 (9.0)	0 (0)	8 (67)	4 (33)

*LC, lymphocytic colitis; LC-HR, lymphocytic colitis in histological remission; CC, collagenous colitis; CC-HR, collagenous colitis in histological remission; UC, ulcerative colitis*.

Three controls received immunomodulatory treatment at the time of serum sample collection (betamethasone, *n* = 1; sulfasalazine, *n* = 1; methotrexate, *n* = 1) for the indications rheumatoid arthritis (*n* = 2) and temporary treatment for a possible allergic reaction (*n* = 1). Serum samples were collected at Skåne University Hospital and Linköping University Hospital, Sweden and stored at −80°C until use.

#### Biopsy Samples

One hundred and twenty-nine patients were originally included in the biopsy group, however seven were excluded due to unsuccessful protein preparations or low counts (<10) in the Luminex analysis. After exclusion, our patient population consisted of 120 patients, as two biopsies were taken from the same patient in active disease and another patient had biopsies taken both when in remission and when in active disease resulting in a total of 122 samples (Figure 1B and Table 1). Histological remission (HR) means that patients had a previously histopathologically confirmed diagnosis of MC, clinically in active disease but were histologically in remission. Exclusion criteria for all patients were prior history of CD and/or clinical signs of gastrointestinal infection, ischemic colitis, or neoplastic disease. The controls were divided into two groups depending on the presence/absence of diarrhea: healthy controls and diarrhea controls. All controls had non-inflamed colons and indications for colonoscopy included clinical symptoms or abnormal radiological findings with suspicion of malignancy. Indications for immunomodulatory treatment in the four controls included psoriatic arthritis, rheumatoid arthritis, polymyalgia rheumatica, and asthma. Information regarding immunomodulatory treatments of patients is available in Table 2. Five UC patients with active disease underwent diagnostic colonoscopies due to new symptoms and therefore did not receive any steroid treatment prior to the time of colonoscopy. The mean Mayo score of UC patients with active disease was 1.7 (SD 0.7) and UC patients in remission had a mean Mayo score of 0 (SD 0). Biopsies were taken with a standard forceps from the hepatic flexure in controls, diarrhea controls, and patients with MC, whereas in UC patients, biopsies were obtained from the macroscopically abnormal areas of the (distal) colon. Usually, three biopsies were collected from each locality and patient. All biopsies were collected at Örebro University Hospital, Sweden and stored at −80°C in RNAlater prior to use.

**Table 2:** Immunomodulatory treatments of patients in the colonic biopsy group.

**Diagnosis**	**Immunomodulatory treatment**		
	**Yes** ***n*** **(%)**	**Medication(s), number of patients**	**No** ***n*** **(%)**
Control (*n =* 36)	4 (11)	Betamethasone, *n =* 1; methotrexate, *n =* 1; methotrexate, prednisolone, and sulfasalazine, *n =*1; prednisolone, *n =* 1	32 (89)
Diarrhea control (*n =* 22)	0 (0)	N/A	22 (100)
Active LC (*n =* 9)	0 (0)	N/A	9 (100)
LC-HR (*n =* 6)	1 (17)	Budesonide, *n =* 1	5 (83)
Active CC (*n =* 16)	4 (25)	Budesonide, *n =* 3; cyclosporine and prednisolone, *n =* 1	12 (75)
CC-HR (*n =* 6)	2 (33)	Budesonide, *n =* 1; budesonide and sulfasalazine, *n =* 1	4 (67)
Active UC (*n =* 15)	10 (67)	Mesalazine, *n =* 8; mesalazine and methotrexate, *n =* 1; olsalazine, *n =* 1	5 (33)
UC remission (*n =* 12)	6 (50)	Balsalazide, *n =* 1; mesalazine, *n =* 1; olsalazine, *n =* 1; sulfasalazine, *n =* 3	6 (50)

*LC, lymphocytic colitis; LC-HR, lymphocytic colitis in histological remission; CC, collagenous colitis; CC-HR, collagenous colitis in histological remission; UC, ulcerative colitis*.

### Protein Lysate Preparation From Colonic Biopsies

All biopsies were thawed on ice. A 5 mm stainless steel bead (Cat. #69989, Qiagen, Hilden, Germany) was placed into each Eppendorf tube that was to contain a sample and these tubes were then stored at −20°C. One hundred and twenty microliters RIPA buffer (50 mM Tris Base pH 7.4, 50 mM Tris-HCL pH 7.4, 150 mM NaCl, 1 mM EDTA, 1% Triton X-100, 0.1% Sodium deoxycholate) containing 1 mg/ml of protease inhibitor (Cat. #04693116001, cOmplete—Protease Inhibitor Cocktail Tablets, Roche, Basel, Switzerland) was added to each tube together with a biopsy. All tubes were subsequently placed into a TissueLyser LT (Qiagen, Hilden, Germany) at 50 Hz for 5 min, centrifuged at 15,700 g for 5 min at 4°C, after which the supernatant was collected. The Pierce BCA Protein Assay Kit (Cat. #23227, Thermo Fisher Scientific, Waltham, Massachusetts, USA) was used to determine protein concentration according to kit protocol.

### Luminex

Analyte concentrations were quantified using the ProcartaPlex Human Immuno-oncology Checkpoint Marker Panel 1 14-plex kit (Cat. #EPX14A-15803-901, Thermo Fisher Scientific, Waltham, Massachusetts, US) for the analytes BTLA, GITR, HVEM, IDO, LAG-3, PD-1, PD-L1, PD-L2, TIM-3, CD28, CD80, 4-1BB (CD137), CD27, and CTLA-4 (CD152). Milliplex Map Human Immuno-Oncology Checkpoint Protein Panel 2 (Cat. #HCKP2-11K, Millipore, Burlington, Massachusetts, USA) kits were used for the analytes sCD40L, 4-1BBL, APRIL, IL-2Rα, E-cadherin, Galectin-3, MICA, MICB, and BAFF. Protein lysates were analyzed at a concentration of 4 mg/ml and the results are expressed as pg analyte/mg protein. Analysis was otherwise performed according to kit protocol. When possible, a 5-parameter, otherwise a 4-parameter logistic curve fit was used. Results were obtained through comparison to a standard curve of known concentrations for each analyte. The Luminex^®^ 200 instrument (Austin, Texas, USA) and xPONENT 3.1 software were used to obtain all results. All immunomodulatory molecules included in this study are shown in Table 3.

**Table 3:** The immunomodulatory molecules included in this study, grouped according to their function(s) when in membrane-bound form.

**Inhibitory**	**(Co-)stimulatory**	**Both**
BTLA CTLA-4 E-cadherin IDO LAG-3 PD-1 PD-L1 PD-L2 TIM-3	4-1BB 4-1BBL APRIL BAFF CD27 CD28 CD40L Galectin-3 GITR MICA MICB	CD80 HVEM IL-2Rα

*The colors represent the function of the molecule when in its membrane-bound form. Red, inhibitory, green, (co-) stimulatory, and blue, indicates that the molecule is either inhibitory or stimulatory, depending on which receptor the molecule binds to*.

### Statistical Analysis

#### Univariate Analyses

Using GraphPad Prism version 9.1.2 for macOS (20), Kruskal-Wallis omnibus test was first performed on each of the 23 analytes. All *p*-values were corrected for testing of multiple analytes using the Benjamini–Hochberg procedure (21). If corrected *p*-values remained significant, the original false-discovery rate method of Benjamini–Hochberg was performed to correct for multiple comparisons between all groups within each analyte. The corrected *p*-values are reported as q-values. A confidence level of 0.05 was used. The paired serum samples from patients with active CC and in remission after treatment were analyzed using the Wilcoxon matched-pairs signed rank test and *p*-values were corrected for testing of multiple analytes using the Benjamini–Hochberg procedure. Inclusion criteria were defined as at least 66% of samples in at least one patient group being above the detection limit of the assay. Five analytes (CD28, CTLA-4, HVEM, GITR, PD-L1) were excluded from statistical analysis of the serum samples, whereas all analytes were included in the statistical analysis of the colon biopsy samples.

#### randomForest Analyses

To complement the univariate statistical analysis and to investigate whether a predictive model for potential clinical use could be developed, randomForest analyses were performed for serum samples and biopsies, respectively. R v.4.0.3 (22) and the package randomForest v.4.6-14 (23) were used for this. All R scripts used are available as Supplementary Files 1–3. The R files are packaged as zip archives that can be opened with e.g., Winzip or 7zip. They are plain text files that can be read in any text editor. If a specific editor cannot open them, the file name extension can be changed from “.R” to “.txt.”

As multivariate analyses are sensitive to small sample sizes, we decided to compare control samples (“CTRL,” normal and diarrhea controls) with a group consisting of patients with MC (“MC,” including collagenous and lymphocytic colitis, both with active disease and in histological remission). Patients with UC constituted a smaller group with a heavily skewed sex distribution and were therefore excluded. Predictor variables (analytes) with a level of missing data larger than 10% were excluded from analysis, as were predictors representing analytes with a large proportion of values below the limit of detection (CD28, HVEM, GITR, and PD-L1 for serum samples, but none for biopsies). Patients with values representing outliers in any predictor (as assessed by the Dixon-Reed method) were excluded. As there were only two biopsy samples from men with MC, this randomForest analysis was based on women only. In the remaining samples, missing data was imputed using the randomForest package's “na.roughfix” function. The sample size for serum samples was *N* = 72, and for biopsies *N* = 93. A summary of sample sizes is presented in Table 4, and the two cleaned datasets are available as Supplementary Files 4, 5.

**Table 4:** Sample size and patient demographics for serum and colonic biopsies included in the multivariate randomForest analyses.

**Serum**		**Biopsies**	
**Diagnosis**	***n*** **(%)**	**Diagnosis**	***n*** **(%)**
CTRL	22 (31)	CTRL	56 (60)
MC	50 (69)	MC	37 (40)
**Sex**		**Sex**	
Female	57 (79)	Female	93 (100)
Male	15 (21)	Male	0 (0)
**Serum**	**Sex:** ***n*** **(%)**		
**Diagnosis**	**Female**	**Male**	
CTRL	17 (77)	5 (23)	
MC	40 (80)	10 (20)	

For serum samples and biopsies, respectively, a nested cross-validation was performed to find the best value of the parameter mtry (the number of predictor variables to choose from when selecting a predictor to use for each node in the decision trees that make up the randomForest) and to evaluate the expected performance of the forest in classifying unseen samples. A custom procedure was used for this (see Supplementary Files 1, 2) to allow a leave-one-out approach in the outer loop of cross-validation, as the randomForest R package does not allow this, while using a five-fold cross-validation in the inner loop. The value of mtry that had the lowest cross-validated balanced error rate (arithmetic mean of the error rate of controls and of MC samples) was used to build a forest based on the entire dataset. A forest size of 300 trees was used for cross-validation and building of the final randomForest models.

### Ethical Considerations

Ethical approval was granted by all necessary regional ethical committees. Collection and usage of the biopsies was authorized by the Regional Ethics Review Board of Uppsala (2008-10-15, ethical approval ID #008/278). For serum samples, approval was obtained from the Regional Ethics Review Boards of Lund (2006-06-29, ID #276/2006 and 2012-01-26, ID #2012/32) and Linköping (2012-09-05, ID #2015/216-3108, and 2015-02-05, ID #2015/31-31). All patient data was pseudonymized and handled in accordance with GDPR.

## Results

### Significantly Decreased Concentrations of Soluble Inhibitory as Well as Stimulatory Biomarkers in Serum of CC and LC Patients

**Figure 2:**
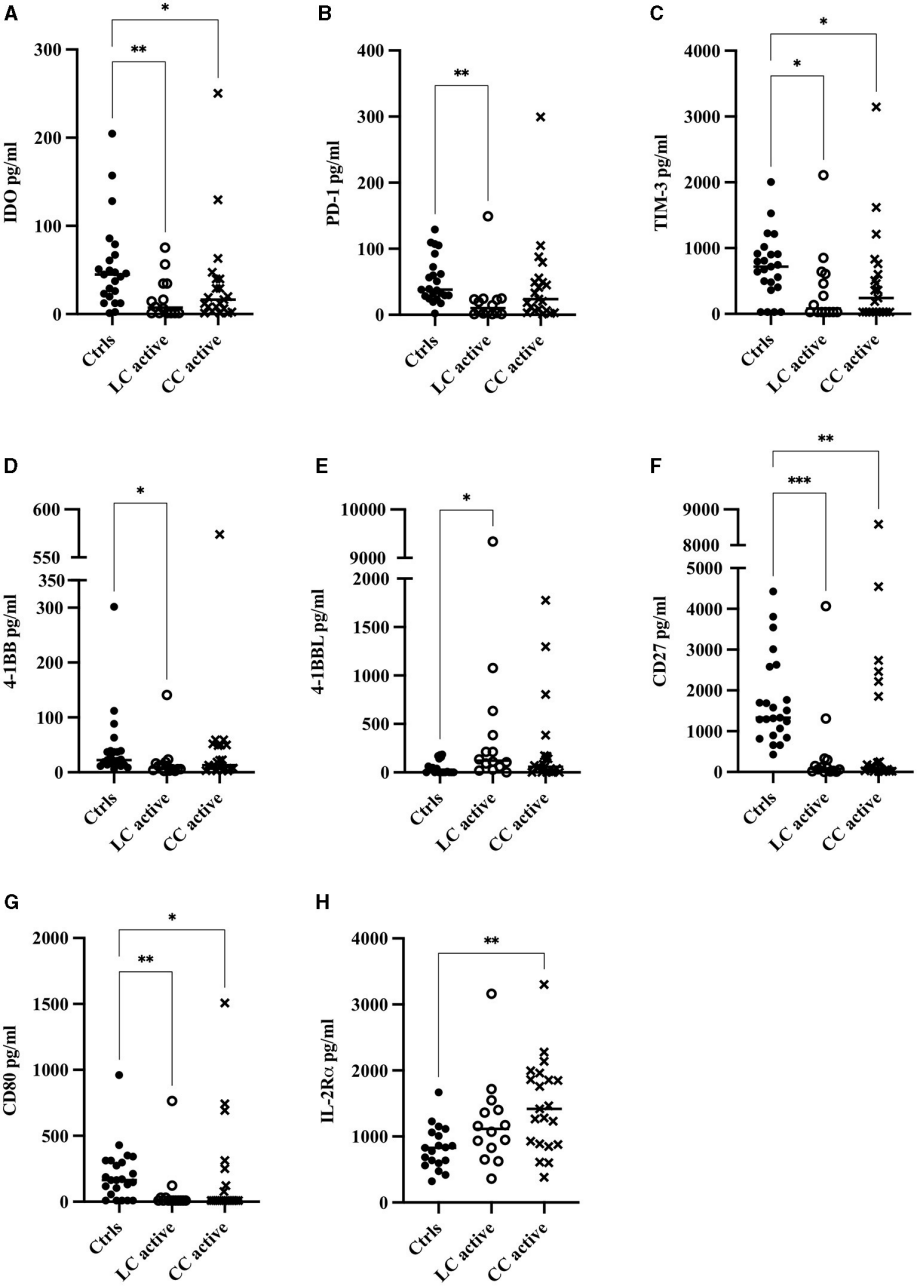
Serum concentrations in pg/ml of all analytes that were significantly altered: **(A)** IDO, **(B)** PD-1, **(C)** TIM-3, **(D)** 4-1BB, **(E)** 4-1BBL, **(F)** CD27, **(G)** CD80, **(H)** IL-2Rα in patients with active lymphocytic colitis (LC) or active collagenous colitis (CC) compared to controls. Each point represents one patient, and the median is shown as a horizontal line. Q-values obtained from performing the original false-discovery rate method of Benjamini–Hochberg are shown as ^*^q < 0.05, ^**^q < 0.01, ^***^q < 0.001.

In MC patients with active disease, significantly decreased levels of the soluble inhibitory molecules IDO and TIM-3 (Figures 2A,C), as well as stimulatory CD27 and CD80 (Figures 2F,G) were seen compared to controls. Significantly decreased levels of soluble PD-1 and 4-1BB were seen in LC but not CC patients with active disease when compared with controls (Figures 2B,D). In contrast, levels of the soluble (co-)stimulatory molecule 4-1BBL in LC patients and soluble IL-2Rα in CC patients were increased compared with controls (Figures 2E,H and Table 5). No statistically significant changes were seen in serum levels of the remaining 10 analytes (Table 5).

**Table 5:** Summary of changes in all analytes in serum and in biopsies from patients with lymphocytic colitis (LC) or collagenous colitis (CC) with active disease and in histological remission (HR).

	**Serum**		**Protein lysates from colonic biopsies**					
**Analyte**	**LC active vs. ctrls**	**CC active vs. ctrls**	**LC active vs. ctrls**	**LC-HR vs. ctrls**	**LC-HR vs. LC active**	**CC active vs. ctrls**	**CC-HR vs. ctrls**	**CC-HR vs. CC active**
**Inhibitory**								
BTLA	▽^1^	=^3^	▽	▽	(▽)^2^	(△)	(▽)	▽
CTLA-4	N/A	N/A	(△)	(▽)	▽	▲ ^a^*q=0.0223*, ^b^*q=0.0178*	▽	▼ *q=0.0185*
E-cadherin	▽	▽	(▽)^a^	▽^a^	▽	▽^a^	▽	▽
IDO	▼ *q=0.0063*	▼ *q=0.0430*	▽	▽	▽	▽	▼^b^ *q=0.0493*	▼ *q=0.0493*
LAG-3	▽	△	▽	▽	▽	▽	▽	▽
PD-1	▼ *q=0.0012*	▽	(△)	(▽)	▽	▲ ^a^*q=0.0207*, ^b^*q=0.0089*	(▽)	▼*q*=*0.0189*
PD-L1	N/A	N/A	(▽)	(▽)	(△)	▲^b^ *q=0.0204*	(▽)	(▽)
PD-L2	▽	(▽)	▲ *q=0.0090*	△	(▽)	▲ ^a^*q=0.0297*, ^b^*q=0.0327*	▲ *q=0.009*	(△)
TIM-3	▼ *q=0.0121*	▼ *q=0.0121*	▼^a^ *q=0.0072*	▼^a^ *q=0.0022*	▽	▽	▼^a^ *q=0.0022*	▽
**(Co)-stimulatory**								
4-1BB	▼ *q=0.0106*	▽	▲^a^ *q=0.0441*	▽^a^	▽	▲ ^a^*q=0.0025*, ^b^*q=0.0047*	▽	▼ *q=0.0047*
4-1BBL	▲ *q=0.0160*	△	(△)	(△)	(△)	(△)	△	△
APRIL	(▽)	(▽)	=^a^	(▽)^a^	(▽)	▲^b^ *q=0.0029*	(▽)^a^	▽
BAFF	(▽)	(▽)	△	▽	▽	▲^b^ *q=0.0326*	▽	▽
CD27	▼ *q=0.0004*	▼ *q=0.0028*	▼^a^ *q=0.0266*	▽	△	▽	▼^a^*q=0.0130*, ^b^*q=0.0325*	▼*q=0.0353*
CD28	N/A	N/A	▽	▼^a^ *q=0.0426*	▽	▽^a^	▽	▽
CD40L	(▽)	(▽)	△	(▽)	▽	(▽)	△	△
Galectin-3	(△)	(△)	△	(▽)	▽	△	▽	▽
GITR	N/A	N/A	▽	▽	(▽)	▽	▽	▽
MICA	△	=	(△)	(▽)^a^	(▽)	(▽)	△	△
MICB	(▽)	(▽)	△	△	(△)	▽	△	△
**Both**								
CD80	▼ *q=0.0083*	▼ *q=0.0182*	▽	▽	▽	▽	▽	▽
HVEM	N/A	N/A	(▽)	▽^b^	(▽)	(▽)	▽	▽
IL-2Rα	△	▲ *q=0.0018*	△	(▽)	▽	▲ ^a^*q=0.0021*, ^b^*q=0.0001*	(△)	▽

1 *Downward arrows (▽▼) indicate a decrease, upward arrows (△▲) indicate an increase in the analyte compared to non-inflamed controls. Filled arrowheads (▲▼) indicate a statistically significant finding in that group, empty arrows (▽△) denote non-significant findings*.

2 *Brackets(()) indicate that the difference was subtle*.

3 *The equals sign (=) denotes that the medians of those groups were equal*.

a *denotes a comparison to controls*;

b *denotes a comparison to diarrhea controls. Unless otherwise stated, a comparison to controls refers to both control groups. N/A means that the analyte failed to meet inclusion criteria (defined as at least 66% of samples in at least one patient group being above the detection limit of the assay) and were thus excluded from analysis. Q-values obtained from performing the original false-discovery rate method of Benjamini–Hochberg are shown*.

#### Budesonide-Induced Remission in CC Patients Is Associated With Significantly Decreased Serum Levels of BAFF and IL-2Rα

**Figure 3:**
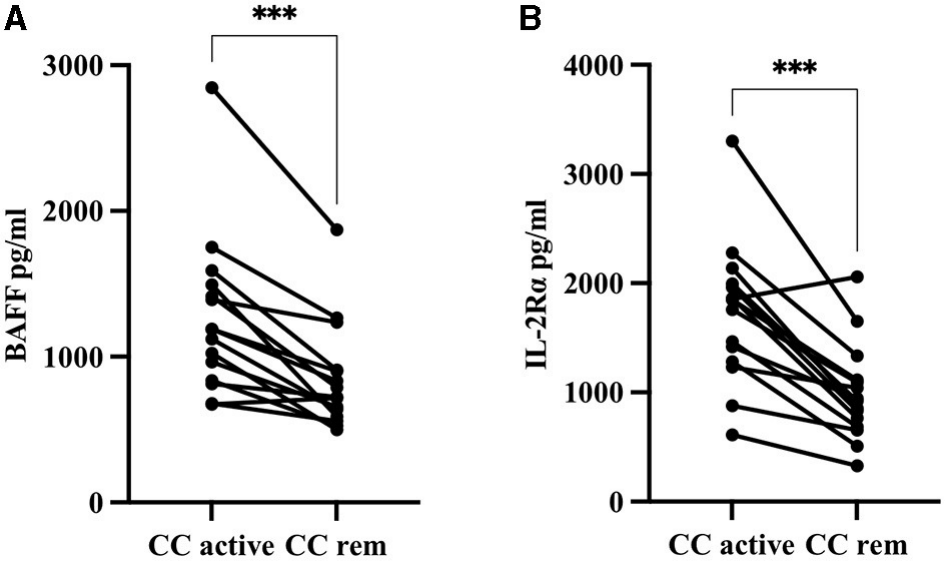
Serum concentrations in pg/ml of **(A)** BAFF and **(B)** IL-2Rα in 15 collagenous colitis (CC) patients with active disease compared to levels in the same patients after budesonide-induced remission. ^***^p < 0.001.

We next compared serum levels of 4-1BB, 4-1BBL, APRIL, BAFF, BTLA, CD27, CD40L, CD80, E-cadherin, Galectin-3, IDO, IL-2Rα, LAG-3, MICA, MICB, PD-1, PD-L2, and TIM-3 in 15 CC patients when in active, untreated disease with levels in the same patients after treatment with budesonide. The subtle decrease in BAFF seen in patients with active CC compared to controls (Table 5), was significantly further decreased following treatment with budesonide, compared to the same patients with active CC (*p* = 0.0001, Figure 3A). IL-2Rα levels, being significantly increased in active CC compared to controls (Figure 2H), were likewise significantly reduced in patients in remission compared to the same patients with active disease (*p* = 0.0002, Figure 3B). Still, levels remained higher than in controls (data not shown).

For the remaining analytes, no statistically significant changes were found, but further decreases in the levels of 4-1BB, 4-1BBL, BTLA, CD27, E-cadherin, IDO, and PD-L2 were observed after budesonide treatment. Levels of APRIL, CD40L, Galectin-3, LAG-3, PD-1, and TIM-3 were instead increased in CC patients in remission, whereas CD80, MICA, and MICB remained unchanged following budesonide treatment (data not shown).

### Significantly Increased Levels of Inhibitory as Well as Stimulatory Biomarkers in Colonic Biopsies From CC and LC Patients

**Figure 4:**
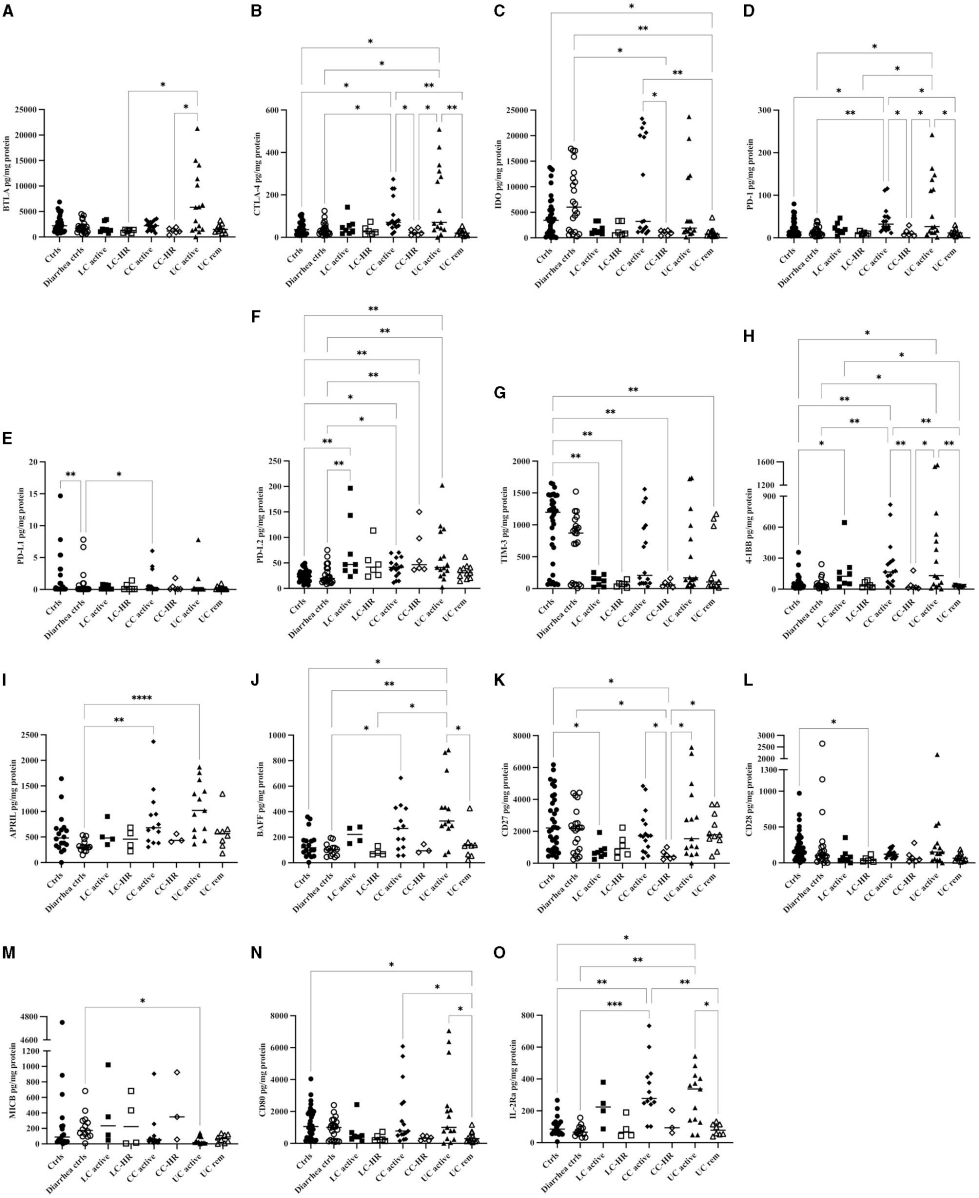
Concentrations in pg/mg protein of all 15 analytes that were significantly altered in protein lysates from colonic biopsies from patients with active lymphocytic colitis (LC) and LC in histological remission (HR), active collagenous colitis (CC) and CC in HR, as well as active ulcerative colitis (UC) and UC in remission compared to controls. **(A)** BTLA, **(B)** CTLA-4, **(C)** IDO, **(D)** PD-1, **(E)** PD-L1, **(F)** PD-L2, **(G)** TIM-3, **(H)** 4-1BB, **(I)** APRIL, **(J)** BAFF, **(K)** CD27, **(L)** CD28, **(M)** MICB, **(N)** CD80, **(O)** IL-2Rα. Each point represents one patient, and the median is shown as a horizontal line. *Q*-values obtained from performing the original false-discovery rate method of Benjamini–Hochberg are shown as ^*^q < 0.05, ^**^q < 0.01, ^***^q < 0.001, ^****^q < 0.0001.

In contrast to the observed results in serum, CC patients with active disease had increased levels of the inhibitory checkpoint molecules CTLA-4, PD-1, PD-L1, and PD-L2, but also (co-)stimulatory 4-1BB, APRIL, BAFF, and IL-2Rα, in colonic biopsies compared to controls (Figure 4 and Table 5).

In both LC and CC patients with active disease, levels of PD-L2 were increased compared to both control groups and it was also significantly increased in CC-HR compared to controls (Figure 4F). 4-1BB levels were increased compared to both control groups in CC and to healthy controls in LC (Figure 4H). Patients with active CC had significantly higher levels of 4-1BB compared to CC-HR (Figure 4 and Table 5).

Patients with active CC, but not LC, had significantly increased levels of the inhibitory checkpoint molecules CTLA-4 and PD-1 compared to both control groups as well as to CC-HR patients (Figures 4B,D). PD-L1 in patients with active CC was significantly increased compared to diarrhea controls (Figure 4E). The stimulatory biomarkers APRIL and BAFF were also increased compared to diarrhea controls in patients with active CC, but not LC (Figures 4I,J). IL-2Rα, exclusively expressed on activated T lymphocytes as well as regulatory T cells, was significantly increased in patients with active CC in comparison to both control groups (Figure 4O). With the exception of PD-L2, levels of all these analytes in LC patients with active disease were lower than in CC patients. Likewise, levels in patients in remission were generally lower than in patients with active disease (Figure 4 and Table 5).

#### Decreased Levels of TIM-3, CD27, and CD28 in Colonic Biopsies of LC Patients, as Well as IDO in CC-HR Patients

LC patients with active disease had significantly decreased levels of TIM-3 (Figure 4G), an inhibitory biomarker expressed on T helper 1 cells and the co-stimulatory molecule CD27 in comparison to healthy controls (Figure 4K). Although levels in CC patients with active disease were also decreased compared to controls, this did not reach statistical significance. In addition, TIM-3 was significantly decreased in both CC-HR and LC-HR compared to healthy controls. CD27 levels were significantly reduced in CC-HR compared to both control groups as well as CC patients with active disease (Figure 4 and Table 5).

The co-stimulatory molecule CD28 was generally decreased in MC patients compared to one or both control groups, but only reached statistical significance in LC-HR patients compared to healthy controls (Figure 4L). Likewise, IDO was decreased in MC patients compared to both control groups, but significantly decreased only in CC-HR patients compared to diarrhea controls. In CC patients with active disease, almost half had very high levels of IDO, even higher than in controls and the whole group had significantly enhanced levels compared to CC-HR patients (Figure 4C).

The remaining 11 analytes: 4-1BBL, BTLA, CD40L, CD80, E-cadherin, Galectin-3, GITR, HVEM, LAG-3, MICA, and MICB in active MC all failed to reach significance when compared to controls. However, in CC patients, there was a trend of increased levels of 4-1BBL, BTLA, and Galectin-3, and insignificantly decreased levels of the other aforementioned analytes. In LC patients, 4-1BBL, CD40L, Galectin-3, MICA, and MICB levels were increased whereas BTLA, CD28, CD80, E-cadherin, GITR, HVEM, IDO, and LAG-3 were lower than in controls (data not shown).

#### No Significant Difference in Levels of Colonic Immunomodulatory Biomarkers in MC Patients Compared With UC Patients

As immune changes in UC patients are hitherto more thoroughly described and given the higher degree of colonic inflammation in these patients, we also compared our findings in colonic biopsies from MC patients with those in UC patients in active disease as well as in remission.

There were no significant changes in any of the 23 analytes between active MC and active UC, nor between UC patients in remission and LC-HR or CC-HR with the exception of CD27, being significantly higher in UC-R compared to CC-HR patients (Figure 4K and Table 6). Although not statistically significant, levels of E-cadherin, 4-1BBL, CD40L, Galectin-3, and MICB were higher in patients with active MC compared with active UC (Table 6). In patients with active CC, levels of IDO, TIM-3, and 4-1BB were insignificantly increased in comparison to patients with active UC.

**Table 6:** Summary of changes in all analytes in biopsies from patients with ulcerative colitis (UC) with active disease and in remission, compared with control groups and patients with lymphocytic colitis in histological remission (LC-HR) as well as patients with collagenous colitis in histological remission (CC-HR).

**Analyte**	**UC active vs. CC active**	**UC active vs. LC active**	**UC active vs. ctrls**	**UC rem vs. CC rem**	**UC rem vs. LC rem**	**UC rem vs. ctrls**	**UC rem vs. UC active**
**Inhibitory**							
BTLA	Δ^1^	Δ	Δ	Δ	Δ	▽	▽
CTLA-4	(▽)^2^	Δ	▲ ^a^*q=*0.0412, ^b^*q=*0.0200	(▽)	(▽)	▽	▼ *q=*0.0039
E-cadherin	▽	▽	▽	△	△	△	△
IDO	▽	△	▽	▽	▽	▼ ^a^*q=0.0493*, ^b^*q=0.0068*	▽
LAG-3	(▽)	△	▽	(△)	(△)	▽	▽
PD-1	(▽)	△	▲^b^ *q=0.0189*	(Δ)	(Δ)	(▽) ^a^	▼ *q=0.0189*
PD-L1	(▽)	(△)	(▽)	(▽)	(▽)	(▽)	(▽)
PD-L2	(▽)	(▽)	▲ ^a^*q=0.0090*, ^b^*q=0.0090*	▽	▽	△	▽
TIM-3	▽	△	▽	△	△	▼^a^ *q=0.0075*	▽
**(Co)-stimulatory**							
4-1BB	▽	(△)	▲ ^a^*q=0.0146*, ^b^*q=0.0206*	(△)	(▽)	▽	▼ *q=0.0047*
4-1BBL	▽	▽	(▽)	▽	▽	(Δ)^a^	(△)
APRIL	△	△	▲^b^ *q < 0.0001*	△	△	△	▽
BAFF	△	△	▲ ^a^*q=0.0148*, ^b^*q=0.0031*	△	△	△	▼*q=0.0326*
CD27	▽	△	▽	▲ *q=0.0328*	△	▽	△
CD28	△	△	(▽) ^a^	(△)	△	▽	▽
CD40L	▽	▽	▽	▽	▽	▽	(▽)
Galectin-3	▽	▽	△^a^	△	△	△	△
GITR	(△)	△	(▽)	▽	(▽)	▽	▽
MICA	(△)	▽	▽^a^	▽	(▽)	(▽) ^a^	(△)
MICB	▽	▽	▼^b^ *q=0.0156*	▽	▽	▽	△
**Both**							
CD80	△	△	▽^a^	▽	△	▼^a^ *q=0.0439*	▼ *q=0.0439*
HVEM	△	△	△	△	△	△	(△)
IL-2Rα	△	△	▲ ^a^*q=0.0236*, ^b^*q=0.0021*	▽	△	(▽) ^a^	▼*q=0.0236*

1 *Downward arrows (▽▼) indicate a decrease, upward arrows (△▲) indicate an increase in the analyte compared to non-inflamed controls. Filled arrowheads (▲▼) indicate a statistically significant finding in that group, empty arrows (▽△) denote non-significant findings*.

2 *Brackets(()) indicate that the difference was subtle*.

a *denotes a comparison to controls*;

b *denotes a comparison to diarrhea controls. Unless otherwise stated, a comparison to controls refers to both control groups*.

*Q-values obtained from performing the original false-discovery rate method of Benjamini–Hochberg are shown*.

Decreased levels of inhibitory and (co-)stimulatory biomarkers were typically observed in patients with UC in remission compared to those with active disease and this was statistically significant for the analytes CTLA-4, PD-1, 4-1BB, BAFF, CD80, and IL-2Rα (Figure 4 and Table 6).

Compared with controls, UC patients with active disease had increased levels of the inhibitory biomarkers CTLA-4, PD-1, and PD-L2, and increases in the (co-)stimulatory molecules 4-1BB, APRIL, BAFF, as well as in IL-2Rα, were also seen. Stimulatory MICB was instead significantly decreased in patients with active UC in comparison to controls (Figure 4 and Table 6).

### randomForest Analysis

For serum samples, a randomForest predictive model was obtained that had an error rate of 0%, a balanced error rate of 0%, and class-specific error rates of 0% for both CTRL and MC. The corresponding cross-validated (CV) error rates were 13.9% (overall), 13.8% (balanced), 13.6% (for CTRL) and 14% (for MC) (data not shown). The predictors (analytes) with the highest variable importance, as assessed by mean decrease in accuracy when each predictor is removed from analysis, were CD27 (41.1% decrease in accuracy), IL-2Rα (7.0%), CD80 (4.3%) and CD137 (4.3%). Variable importance was also assessed by mean decrease in Gini impurity (the larger the decrease, the more important the variable). Results are shown in Table 7.

**Table 7:** Summary of predictors (analytes) with the highest variable importance in serum, assessed both in terms of mean decrease in accuracy and mean decrease in Gini impurity.

**Predictor**	**Mean decrease accuracy**	**Predictor**	**Mean decrease Gini**
CD27	41.07	CD27	19.21
IL-2Rα	7.04	4-1BB	2.22
CD80	4.34	BTLA	1.38
4-1BB	4.31	CD80	1.36
BTLA	2.21	IL-2Rα	1.32
APRIL	2.20	MICB	0.92
MICA	1.71	BAFF	0.58
IDO	1.17	CD40L	0.56
E-cadherin	1.15	MICA	0.41
MICB	0.78	PD-L2	0.39
Age	0.27	Age	0.34
BAFF	0.26	APRIL	0.26
TIM-3	−0.01	Galectin-3	0.25
LAG-3	−0.44	E-cadherin	0.22
Galectin-3	−0.48	TIM-3	0.21
PD-1	−0.59	PD-1	0.20
CD40L	−0.68	CTLA-4	0.15
PD-L2	−1.38	LAG-3	0.11
4-1BBL	−1.46	IDO	0.08
CTLA-4	−2.59	4-1BBL	0.05

For biopsies, the predictive model obtained had an error rate of 0%, a balanced error rate of 0%, and class-specific error rates of 0% for both CTRL and MC. The corresponding cross-validated error rates were 14.0% (overall), 14.8% (balanced), 10.7% (for CTRL), and 18.9% (for MC) (data not shown). The predictors with the highest variable importance were PD-L2 (14.0% decrease in accuracy), TIM-3 (12.1%), IDO (11.6%), 4-1BB (8.0%), and LAG-3 (7.6%). These results are shown Table 8, along with the reduction in Gini impurity.

**Table 8:** Summary of predictors (analytes) with the highest variable importance in biopsies, assessed both in terms of mean decrease in accuracy and mean decrease in in Gini impurity.

**Predictor**	**Mean decrease accuracy**	**Predictor**	**Mean decrease Gini**
PD-L2	14.05	PD-L2	8.34
TIM-3	12.12	TIM-3	5.98
IDO	11.61	4-1BB	5.16
4-1BB	7.97	IDO	5.04
LAG-3	7.55	CD80	3.04
CTLA-4	4.73	CTLA-4	2.79
CD80	4.20	LAG-3	2.78
BTLA	3.98	CD28	1.98
CD27	3.87	PD-1	1.91
CD28	3.16	CD27	1.88
GITR	2.53	BTLA	1.67
PD-1	2.29	Age	1.62
HVEM	−0.38	GITR	0.95
Age	−3.04	HVEM	0.78

## Discussion

In summary, in serum, decreases in the inhibitory molecules TIM-3, PD-1, and IDO; the (co-) stimulatory molecules 4-1BB and CD27; and CD80 (inhibitory when bound to CTLA-4) were seen in MC patients compared with controls whereas levels of 4-1BBL and IL-2Rα were instead increased in MC patients. In contrast, in biopsies, levels of PD-L2 and 4-1BB were increased in both MC and UC patients with active disease. Furthermore, in biopsies from CC and UC but not LC patients with active disease, increases in CTLA-4, PD-1, APRIL, BAFF, and IL-2Rα were seen compared with controls. Levels of CD27 and TIM-3 were however decreased in biopsies from MC patients in comparison to controls whereas levels of MICB were decreased in patients with active UC compared with controls. Some of these molecules were also found to be among the most important for the distinction between MC and CTRL in the randomForest analysis.

CD8^+^ T cells play an important role in anti-tumor defense through e.g., secretion of cytotoxic substances that induce apoptosis in target cells (24). Numerous studies have found that, in contrast to classical forms of IBD, CD8^+^ rather than CD4^+^ T cells seem to be the more dominant cell type in patients with MC (25–27). Although patients with MC have a lower risk of developing CRC compared with patients with UC and the general population, MC patients have an increased risk for other malignancies, e.g., lung cancer (8, 28), indicating that the protective effect may be local, e.g., through increased activity of CD8^+^ CTLs due to the colitis. 4-1BB, found to be significantly increased in biopsies from patients with active MC and UC compared to controls and most highly expressed in active CC, is a co-stimulatory receptor present on activated T cells that induces a stronger stimulatory response in CD8^+^ compared to CD4^+^ T cells (29). In partial contrast with our results, a previous study has reported increased expression of 4-1BB in lamina propria T cells in CD patients compared to UC patients and controls, as well as decreased 4-1BB mRNA levels in UC patients (17). However, mRNA levels do not always reflect protein expression due to complex regulatory mechanisms (30). 4-1BB has an important role in aiding the survival of CD8^+^ T-cells through activation of anti-apoptotic pathways involving Bcl-x_L_ and Bfl-1 (31). Results from a mouse model of CD showed that treatment with anti-4-1BB monoclonal antibodies seemed to have an anti-inflammatory effect in the colon and increased proportions of splenic CD8^+^ T cells (32). Interestingly, we observed decreased levels of 4-1BB in serum in patients with active LC compared to controls and it is possible that the soluble form of 4-1BB can act as a decoy molecule and then instead have an inhibitory effect (33).

APRIL (a proliferation-inducing ligand) and BAFF (B cell activating factor), expressed on myeloid cells, are involved in the differentiation and survival of B cells (34). Studies have documented increased expression of BAFF in colonic tissue, feces, and serum from patients with IBD compared with healthy controls and highlight BAFF's potential as a marker of disease activity (35–37). Our findings of significantly increased levels of APRIL and BAFF in biopsies from patients with active CC or UC, with the same trend seen in active LC, is in line with previous findings in IBD. Soluble forms of APRIL and BAFF seem to have similar functions as their membrane-bound forms. However, it should be noted that soluble forms of the receptors for APRIL and BAFF (BAFFR, TACI, and BCMA) may act as decoys and thus inhibit the expected stimulatory effects (34).

We found significantly decreased levels of CD27, a stimulatory molecule on T cells, in serum from patients with active MC, as well as in biopsies from patients with active LC with the same decreasing trend seen in biopsies from patients with active CC and UC. Evidence from two mouse models of colitis suggests that binding of CD27 to its ligand CD70 on antigen-presenting cells plays a role in mediating colitis (38). Soluble CD27 likewise seems to exhibit stimulatory functions. The reduced levels in MC found in this study indicate a negative feedback mechanism, with reduced expression as a result of the inflammation (39).

The MHC class I-related molecules A and B (MICA and MICB) are polymorphic cell surface glycoproteins that are induced on e.g., intestinal epithelial cells following cell stress. These are seen by the receptor NKG2D, expressed on NK cells as well as CD8^+^ TcRαβ^+^ or TcRγδ^+^ T cells (40). It was therefore somewhat surprising to not see any significantly increased expressions of these molecules, but instead significantly reduced colonic expression of MICB in patients with active UC. We found a trend toward increased expression in LC patients, possibly reflecting the high influx of lymphocytes in the epithelium.

The BTLA (B- and T-lymphocyte attenuator) protein is induced on T cells following activation and acts as a ligand for the herpes virus entry mediator (HVEM), resulting in inhibition of T-cell responses and thereby playing an essential immunomodulatory role in different diseases including autoimmune diseases, cancer, transplantation and infection (41). We found trends for reduced levels of BTLA in MC biopsies, but significantly enhanced levels in patients with active UC. It is possible that the enhanced expression in UC reflects the higher degree of inflammation in these patients. The trend for reduced expression of colonic as well as serum BTLA in MC, together with previous reports on a dominance of CD8^+^ T cells in the mucosa of these patients is in line with the findings of BTLA inhibiting human tumor specific CD8^+^ T cells (42). Increased levels of soluble BTLA have been found to correlate with lower survival rates in patients with clear renal cell carcinoma and patients with advanced hepatocellular carcinoma (43, 44).

IDO is an immunosuppressive enzyme found to be more highly expressed in patients with IBD compared to controls and this is thought to signify an attempt to dampen the ongoing inflammation (45, 46). However, IDO-deficient mice have been shown to have ameliorated colitis, associated with significantly reduced expression of members of the TLR-MyD88-NF-kB signaling pathways and significantly reduced levels of pro-inflammatory cytokines, indicating that IDO may also have pro-inflammatory effects on non-T-cells (47). Our findings of significantly decreased serum levels in MC patients with active disease compared to controls, with levels in biopsies also displaying the same trend, are a bit puzzling given that MC patients have ongoing inflammation, albeit milder than in UC (28), and thus increased expression of IDO in MC patients compared with controls would be expected. Surprisingly, UC patients in our study also displayed insignificantly reduced levels of IDO in comparison to both control groups as well as CC but not LC patients with active disease. IDO has inhibitory effects on effector T cells and induces differentiation of regulatory T cells (Treg), likely explaining its observed role in suppressing anti-tumor immunity (48) and its expression in tumor cells in CRC (49). At present, it is unknown whether the decreases in IDO observed in MC patients in this study could be related to the decreased risk of CRC in MC patients.

LAG-3 is an inhibitory immune checkpoint molecule expressed on activated CD4^+^ and CD8^+^ T cells (50). Another study found that LAG-3^+^ lymphocytes were more abundant in the inflamed colon of patients with UC compared with controls but not in peripheral blood from UC patients, suggesting that LAG-3 acts locally in the mucosa (51). Our study, in contrast to this, showed a trend of decreased LAG-3 levels in biopsies from patients with active MC and UC.

PD-1 is an inhibitory receptor expressed on activated T cells that has been reported to be upregulated in IBD (19, 52). In accordance with this, we also found increased expression in the colon of MC and UC patients with active disease. The opposite was seen in serum where levels of PD-1 were decreased. Reviews of the functions of soluble PD-1 (sPD-1) describe the theory that sPD-1 can out-compete membrane-bound PD-1 with regards to binding to its ligands (PD-L1, -L2, and CD80), thus blocking the expected inhibitory effects (15, 53). The reduced serum levels of PD-1 indicate that membrane-bound PD-1 can exert its effects with less competition from sPD-1, thereby exerting more inhibition of the inflammation seen in patients with IBD and MC. In line with our results in MC and UC patients, another study found increased levels of PD-L1 and PD-L2 mRNA in biopsies from UC patients compared with controls (54).

IL-2Rα is expressed on T regulatory cells as well as activated T cells which can shed soluble IL-2Rα (sIL-2Rα). The definitive role of sIL-2Rα has not yet been elucidated, with both agonistic and antagonistic effects being described (55). A study investigating sIL-2Rα in serum of patients with multiple sclerosis suggests that sIL-2Rα is more likely to act as a decoy and bind up IL-2 (56). We demonstrated significantly increased levels of IL-2Rα in CC patients with active disease in both serum and biopsies, as well as in biopsies from patients with active UC, compared to controls. This could indicate a decoy role in serum, inhibiting inflammation by binding up IL-2. This assumption is supported by the significantly reduced levels of sIL-2Rα in CC patients in remission following budesonide treatment. As we cannot distinguish between soluble and membrane-bound IL-2Rα in the colonic biopsies, it is likely that our results reflect enhanced membrane-bound levels in the inflamed mucosa.

The expression of TIM-3, an inhibitory receptor on T cells, was decreased in serum and biopsies from patients with active MC and in biopsies from patients with UC, albeit insignificantly in the latter. Although the function of soluble TIM-3 still remains unclear, there is some evidence that it also acts in an inhibitory manner (57). The reduced levels of TIM-3 in the colonic mucosa are surprising as most inhibitory checkpoints are upregulated during chronic inflammation. Further studies are needed to explain this finding.

CD80 is expressed on dendritic cells as well as activated monocytes and B cells (15) and, in its membrane-bound form, can either be stimulatory if binding to CD28 or inhibitory if binding to CTLA-4. Studies by Scarpa et al. showed that UC patients with dysplasia displayed higher levels of CD80 than those without dysplasia, and that CD80 levels were significantly higher in UC patients with dysplasia compared to those with non UC-related dysplasia (58), suggesting that CD80 may be involved in immune surveillance in UC patients (43, 44). Soluble CD80 has been reported to have stimulatory effects through inhibition of the PD-1/PD-L1 pathway and binding to costimulatory CD28 (59). Findings from our study demonstrate reduced levels of CD80 in serum from MC patients and insignificantly decreased levels in biopsies from both MC and UC patients. This may possibly represent an attempt to downregulate the ongoing inflammation, but further studies are necessary to explain these findings. Whereas, naïve T cells express the costimulatory molecule CD28 and get stimulating signals through binding to its ligands CD80/CD86, the CD28 is exchanged for CTLA-4 upon T cell activation, binding CD80/CD86 with higher affinity and providing down-regulatory signals to the T cell (57, 58, 60, 61). The reduced colonic levels of CD28, together with enhanced levels of CTLA-4 in MC and UC patients compared to controls likely reflect reduced numbers of naïve and increased numbers of activated T cells in the mucosa.

The very low error rates obtained with the randomForest analyses for both serum samples and biopsies demonstrate that the respective predictive models perform well in classifying patient and control samples used in this study. The cross-validated error rates suggest that both models can be expected to perform reasonably well in classifying new samples. Thus, these models have a potential for being clinically useful in detecting microscopic colitis. In this context, serum samples would be preferred over biopsies, being less invasive. The serum model also performed well in classifying both men and women, with error rates of 0 for both sexes, and cross-validated error rates of 15.8% for women and 6.7% for men). For biopsies, the performance of the model in men could not be evaluated, since they were too few to be included in the analysis. If data from more MC patients and controls could be obtained, this model could probably be further improved—perhaps also being able to distinguish between subgroups of microscopic colitis.

In addition to providing potential clinical utility as a diagnostic tool, the variable importance found from the randomForest analyses to some extent support the findings from the univariate analyses; analytes 4-1BB, CD27, CD80, and IL-2Rα again seemed to play an important role in the differences observed in serum between controls and MC patients. In biopsies, IDO, LAG-3, PD-L2, TIM-3, and 4-1BB appeared to be the key distinguishing analytes when comparing controls and patients with MC.

A strength of this study is that levels of inhibitory and stimulatory biomarkers were studied in both serum and biopsies, providing information about the systemic and local immune response in MC patients compared to controls. However, this is also a limitation as we were unable to distinguish between the membrane-bound and soluble forms of these analytes in protein lysates, although most of the protein detected is likely from membrane-bound forms. Another limitation is that serum and biopsy samples were not collected from the same patient population. In addition to this, the diagnoses of MC and UC were not confirmed by a second, independent pathologist. However, all samples were judged based on well-defined international criteria for diagnosis, as part of the routine examination of biopsies by experienced gastroenterology pathologists at the Depts of Pathology at the three centers (Örebro, Linköping, and Malmö) involved in the present study. We were unable to retrieve data on former smoking, and it cannot be ruled out that some of the current non-smokers are previous smokers. In addition to smoking, alcohol consumption is a risk factor associated with the development of CRC (62, 63) and, although findings regarding alcohol intake are inconclusive, have also been found to be associated with MC development (64–66). Alcohol can also affect the immune system so information about alcohol intake would be valuable to include in future studies (63, 67).

This is, to the best of our knowledge, the first analysis of a broad array of checkpoint molecules systemically as well as locally in the colonic mucosa of MC patients, compared to controls as well as UC patients. The findings need to be confirmed in additional cohorts, but nevertheless contribute to the limited field of knowledge about checkpoint molecules and their role as biomarkers in MC and may also contribute to knowledge about possible mechanisms behind the seemingly protective effects of MC against colorectal cancer.

## Data Availability Statement

The original contributions presented in the study are included in the article/Supplementary Material, further inquiries can be directed to the corresponding author/s.

## Ethics Statement

The studies involving human participants were reviewed and approved by the Regional Ethics Review Board of Uppsala (2008-10-15, Ethical Approval ID #**2**008/278) for the biopsy samples. For serum samples, approval was obtained from the Regional Ethics Review Boards of Lund (2006-06-29, ID #276/2006 and 2012-01-26, ID #2012/32) and Linköping (2012-09-05, ID #20**12**/216-31 and 2015-02-**2**5, ID #2015/31-31). The patients/participants provided their written informed consent to participate in this study. Written informed consent was obtained from the individual(s) for the publication of any potentially identifiable images or data included in this article.

## Author Contributions

EH, OH, and AL: design of the study. AM and KS: patient recruitment and data collection serum. JB, AWic, and AL: patient recruitment and data collection biopsies. AWir, AL, EH, and OH: data analysis. AL, EH, and AWir: writing the manuscript. All authors: reviewing of the final manuscript.

## Funding

This work was supported by the Faculty of Medicine and Health, Örebro University (EH) and the Örebro University Hospital Research Foundation; OLL 926161 and OLL-960784 (EH and JB).

## Conflict of Interest

The authors declare that the research was conducted in the absence of any commercial or financial relationships that could be construed as a potential conflict of interest.

## Publisher's Note

All claims expressed in this article are solely those of the authors and do not necessarily represent those of their affiliated organizations, or those of the publisher, the editors and the reviewers. Any product that may be evaluated in this article, or claim that may be made by its manufacturer, is not guaranteed or endorsed by the publisher.

## Abbreviations

APRIL: a proliferation-inducing ligand
BAFF: B-cell activating factor
BTLA: B- and T-lymphocyte attenuator
CC: collagenous colitis
CD: Crohn's disease
CD27: cluster of differentiation 27
CRC: colorectal cancer
CTLA-4: cytotoxic T-lymphocyte-associated antigen 4
E-cadherin: epithelial cadherin
GITR: glucocorticoid-induced tumor necrosis factor receptor-related protein
HR: histological remission
HVEM: herpesvirus entry mediator
IBD: inflammatory bowel disease
IDO: indoleamine 2, 3-dioxygenase
IEL: intraepithelial lymphocyte
IL-2Rα: interleukin-2 receptor alpha chain
LAG-3: lymphocyte-activation gene 3
LC: lymphocytic colitis
MC: microscopic colitis
MICA: MHC class I chain-related protein A
MICB: MHC class I chain-related protein B
PD-1: programmed cell death protein 1
PD-L1: programmed death-ligand 1
PD-L2: programmed death-ligand 2
sCD40L: soluble cluster of differentiation 40 ligand
TIM-3: T-cell immunoglobulin and mucin domain-containing protein 3
UC: ulcerative colitis.
