# Corrigendum: Patients with microscopic colitis have altered levels of inhibitory and stimulatory biomarkers in colon biopsies and sera compared to non-inflamed controls

**DOI:** 10.3389/fmed.2024.1506094

**Published:** 2024-11-12

**Authors:** Alexandra Lushnikova, Johan Bohr, Anna Wickbom, Andreas Münch, Klas Sjöberg, Olof Hultgren, Anders Wirén, Elisabeth Hultgren Hörnquist

**Affiliations:** ^1^School of Medical Sciences, Örebro University, Örebro, Sweden; ^2^Division of Gastroenterology, Department of Medicine, Örebro University Hospital, Faculty of Medicine and Health, Örebro University, Örebro, Sweden; ^3^Department of Gastroenterology and Hepatology in Linköping, and Department of Health, Medicine, and Caring Sciences, Linköping University, Linköping, Sweden; ^4^Department of Clinical Sciences, Lund University, Department of Gastroenterology, Skåne University Hospital, Malmö, Sweden; ^5^Department of Clinical Immunology and Transfusion Medicine, Faculty of Medicine and Health, Örebro University, Örebro, Sweden

**Keywords:** microscopic colitis, colorectal cancer, immune surveillance, immune checkpoints, ulcerative colitis, serum, colonic biopsies

In the published article, reference Rajabian et al. (2019) (54) was not cited in the article. The citation has now been inserted in the **Discussion**, paragraph 9, and should read:

“PD-1 is an inhibitory receptor expressed on activated T cells that has been reported to be upregulated in IBD (19, 52). In accordance with this, we also found increased expression in the colon of MC and UC patients with active disease. The opposite was seen in serum where levels of PD-1 were decreased. Reviews of the functions of soluble PD-1 (sPD-1) describe the theory that sPD-1 can out-compete membrane-bound PD-1 with regards to binding to its ligands (PD-L1, -L2, and CD80), thus blocking the expected inhibitory effects (15, 53). The reduced serum levels of PD-1 indicate that membrane-bound PD-1 can exert its effects with less competition from sPD-1, thereby exerting more inhibition of the inflammation seen in patients with IBD and MC. In line with our results in MC and UC patients, another study found increased levels of PD-L1 and PD-L2 mRNA in biopsies from UC patients compared with controls (54).”

In the published article, there was an error. Two ethical approval IDs and the date of one of the approvals from the Regional Ethics Review Board were stated incorrectly.

A correction has been made to the **Ethics Statement**. This sentence previously stated:

“The studies involving human participants were reviewed and approved by the Regional Ethics Review Board of Uppsala (2008-10-15, Ethical Approval ID #008/278) for the biopsy samples. For serum samples, approval was obtained from the Regional Ethics Review Boards of Lund (2006-06-29, ID #276/2006 and 2012-01-26, ID #2012/32) and Linköping (2012-09-05, ID #2015/216-3108 and 2015-02-05, ID #2015/31-31).”

The corrected sentence appears below:

“The studies involving human participants were reviewed and approved by the Regional Ethics Review Board of Uppsala (2008-10-15, Ethical Approval ID #**2**008/278) for the biopsy samples. For serum samples, approval was obtained from the Regional Ethics Review Boards of Lund (2006-06-29, ID #276/2006 and 2012-01-26, ID #2012/32) and Linköping (2012-09-05, ID #20**12**/216-31 and 2015-02-**2**5, ID #2015/31-31).”

The authors apologize for these errors and state that this does not change the scientific conclusions of the article in any way. The original article has been updated.
